# Self‐Powered SiC‐Based Photoelectrochemical Ultraviolet Photodetectors for Robust Underwater Optical Communication Against Full Aquatic Environments

**DOI:** 10.1002/advs.202513939

**Published:** 2025-10-13

**Authors:** Runchao Dong, Hulin Wang, Jia Zhang, Hongxin Yin, Genqiang Liu, Bartosz Orwat, Beata Luszczynska, Weijun Li, Dongdong Zhang, Huan He, Zhentao Du, Shanliang Chen, Weiyou Yang

**Affiliations:** ^1^ Institute of Micro/Nano Materials and Devices Ningbo University of Technology Ningbo 315211 P. R. China; ^2^ School of Resources, Environment, and Materials Guangxi University Nanning 530004 P. R. China; ^3^ Department of Molecular Physics, Faculty of Chemistry Lodz University of Technology Zeromskiego 116 Lodz 90–924 Poland

**Keywords:** full aquatic applications, heterojunction, localized surface plasmon resonance, photoelectrochemical photodetectors, SiC nanowire arrays

## Abstract

Manipulating the interfacial and surface carrier dynamics within nanostructured semiconductors is pivotal for advancing the electrical and optical performances of photoelectrochemical (PEC) photodetectors (PDs), particularly for durable underwater optical communication systems. Herein, a highly responsive and robust self‐powered PEC UV PDs based on SiC/Au heterojunction nanoarrays is explored, by leveraging the synergistic effect from localized surface plasmon resonance (LSPR) of Au nanoparticles (NPs), heterojunction interfacial engineering, integrated self‐supporting photoelectrode architecture, 1D nanoarray benefits, and inherent chemical stability of SiC. SiC nanowire (NW) arrays are fabricated via anodic oxidation and subsequently decorated with tailored densities of Au NPs using controlled sputtering. The as‐assembled SiC‐based PEC PDs demonstrate exceptional UV (375 nm) photodetection behaviors with rapid response (*τ*_r_/*τ*_d_, 86.5/170.95 ms), high responsivity (*R*_λ_, 1244.95 mA W^−1^), excellent detectivity (*D* ^*^, 6.35 × 10^11^ Jones), and remarkable external quantum efficiency (*EQE*, 412.45%). Moreover, they exhibit broad pH tolerance and excellent long‐term stability against full aquatic environments. This work provides a strategic approach for designing high‐performance self‐powered PEC PDs for reliable underwater optical communication, such as liquid environmental monitoring, ocean exploration, and biomedical detection.

## Graphical abstract

A highly responsive and reliable self‐powered photoelectrochemical ultraviolet photodetector is developed based on integrated self‐supporting SiC/Au heterojunction nanoarrays for underwater optical communication application against full aquatic environments, which exhibits exceptional performance with excellent wide‐pH adaptability, high long‐term stability, and self‐powered operation ability.

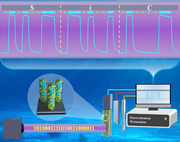

## 1 Introduction

PDs have become vital components driving modern societal development owing to their extensive applications in environmental monitoring, biomedicine, and communication systems.^[^ ^1^, ^2^ ^]^ These devices swiftly and sensitively detect optical signal variations and convert them into digital signals through photocurrent fluctuations to facilitate information transmission and exchange.^[^ ^3^, ^4^, ^5^ ^]^ In addition, their fast response and high sensitivity have made them indispensable in photoelectric imaging technologies.^[^ ^6^ ^]^ UV light, comprising photons with a wavelength of 10–400 nm, possesses high photon energy that renders traditional visible and infrared light detection methods ineffective for UV‐light detection.^[^ ^7^ ^]^ UV PDs have garnered significant attention because of their critical roles in missile warning systems, space exploration, environmental sensing, and digital communication.^[^ ^8^, ^9^ ^]^ With the advancement of high‐sensitivity UV PDs, various semiconductors, such as Ga_2_O_3_,^[^ ^10^ ^]^ GaN,^[^ ^11^, ^12^ ^]^ SiC,^[^ ^13^ ^]^ and ZnO,^[^ ^14^ ^]^ have been explored. However, the existing conventional UV PDs face multiple bottlenecks from technological and material aspects. For instance, their low external quantum efficiency results in insufficient photon utilization. Moreover, their suboptimal detectivity compromises accurate signal identification, making reliable information acquisition impossible in scenarios involving weak‐light detection. In recent years, PEC UV PDs have emerged as a groundbreaking solution with dual significance: their PEC operational mechanism enables real‐time optical monitoring in liquid‐phase environments.^[^ ^15^ ^]^ In addition, their solar‐blind characteristics effectively eliminate interference from visible and near‐UV components in the solar radiation, ensuring precise UV signal detection. Furthermore, as a new type of self‐powered devices, PEC PDs do not require complex lithography processes or external batteries, making them suitable for convenient self‐powered light detection.^[^ ^16^ ^]^ This innovation opens new research directions in PD technology, particularly for underwater environmental monitoring without a power supply.^[^ ^17^ ^]^ However, exploiting superior PEC PDs with self‐power and high reliability characteristics for robust underwater optical communication in full aquatic environments remains a significant challenge.

Two strategies have been adopted to develop excellent PEC UV PDs: optimizing internal structures^[^ ^17^ ^]^ and regulating semiconductor surface properties.^[^ ^18^ ^]^ Both approaches fundamentally rely on the principles of carrier dynamics to control the behavior of the carrier. Surface modification of nanostructured semiconductors and construction of heterojunctions are critical for enhancing PD performance, and the core principle involves introducing functional nanoparticles to optimize the surface and interface properties, improving carrier transport and separation efficiency, accelerating interfacial reaction kinetics, and promoting photoresponses.^[^ ^19^ ^]^ Liu et al.^[^ ^20^ ^]^ employed a surface modification strategy involving Rh‐Cr_2_O_3_ hybrid modified p‐type AlGaN nanowires. The associated solar‐blind PEC PD presented remarkably improved *EQE* from 28.8% to 86.7%. As a typical third‐generation semiconductor, SiC exhibits numerous unique advantages in optoelectronics. SiC has a wide bandgap that results in effective resistance to high‐temperature and high‐radiation environments, and its robust chemical stability ensures reliable performance under complex conditions.^[^ ^13^ ^]^ These characteristics are highly compatible with the application requirements for the stable operation of UV PDs in harsh underwater environments, endowing SiC PEC UV PDs have significant practical value.^[^ ^21^ ^]^ The construction of surface‐loaded noble metals and heterojunctions can effectively adjust the surface properties of wide‐bandgap semiconductors, thereby accelerating the kinetics of interfacial reactions and promoting light response.^[^ ^15^ ^]^ Although work has been explored for the study of wide bandgap semiconductor PEC UV PD composite structure engineering,^[^ ^22^ ^]^ there is still no work focused on the exploration of PEC UV PD based on noble metal modified SiC nanostructures.

This work developed a high‐responsivity and high‐reliability self‐powered PEC UV PD for underwater optical communication in a full aquatic environment based on self‐supporting photoelectrodes comprising integrated SiC/Au heterojunction NW arrays. The SiC NW arrays were fabricated via a two‐step anodic oxidation method, where the oxidized nanoarrays and unoxidized SiC wafer were retained as a whole single‐crystalline integrated self‐supporting configuration. This effectively eliminated the problems caused by interfacial contact between the photoactive layer and current collector that occur commonly in traditional PEC PDs. The obtained Au NPs that deposited on the SiC NWs surface by magnetron sputtering exhibited an excellent LSPR effect, significantly enhancing the carrier transport properties of the photoelectrodes. Additionally, the Au NPs optimized the surface chemical reaction active sites, enabling more efficient redox reactions and further facilitating the PEC process. Consequently, the developed SiC/Au PEC UV PD exhibited exceptional photodetection behaviors with a rapid *τ*_r_/*τ*_d_, high *R*_λ_, remarkable *D* ^*^, and record‐breaking *EQE* of 86.5/170.95 ms, 1244.95 mA W^−1^, 6.35 × 10^11^ Jones, and 412.45% under 375‐nm UV‐light illumination. These values significantly surpassed those of the original SiC nanoarrays. Remarkably, the device also exhibited excellent wide‐pH adaptability, high long‐term stability, and self‐powered operation ability. This work provides a novel and reliable technical concept for developing highly responsive and reliable optical communication systems that can operate in harsh full aquatic environments.

## 2 Results and Discussion

**Figure 1:**
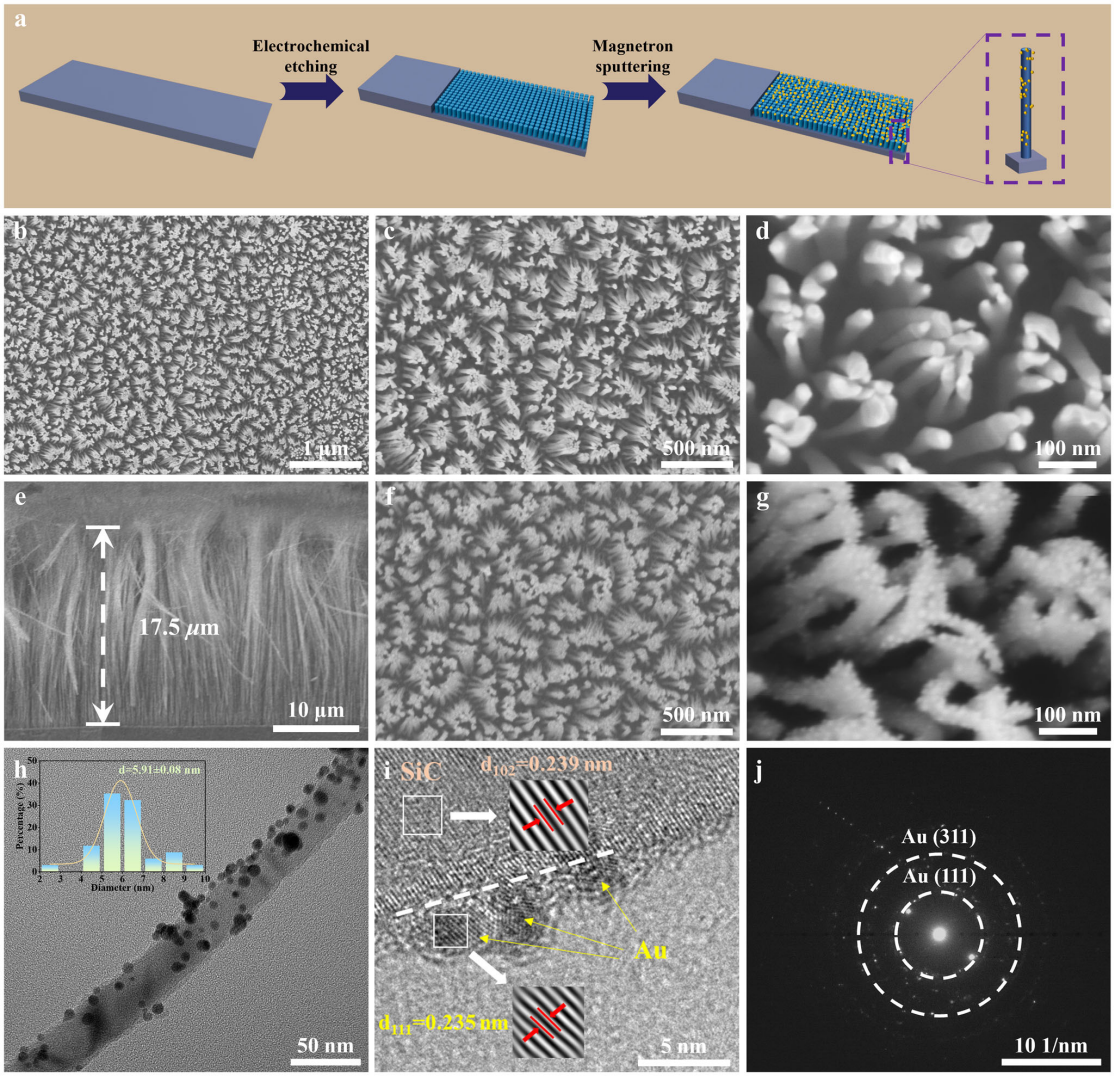
a) Schematic of the fabrication process for the integrated self‐supporting SiC/Au composite NW arrays. b–d) Top‐view SEM images of etched SiC NW arrays at different magnifications. e) Cross‐sectional SEM image of SiC NW arrays. f,g) Top‐view SEM images of SiC/Au composite NW arrays at different magnifications. h) The typical TEM image of a SiC/Au composite NW. The inset shows the size distribution of Au NPs on the surface of SiC NW. i) The HRTEM, and j) SAED of the SiC/ Au composite NW.

**Figure** **1**a illustrates the representative fabrication process of the integrated self‐supporting SiC/Au composite NW array photoelectrodes. First, the single‐crystal SiC wafer was subjected to a two‐step anodic oxidation process. The resulting single‐crystal SiC wafer was used as an integrated self‐supporting photoelectrode, where the unetched portion acted as the current collector and the electrochemically etched nanostructures functioned as the photoactive layer. Subsequently, Au NPs were deposited onto the surface of the etched SiC nanostructures via ion sputtering. Integrated self‐supporting SiC/Au composite nanostructured photoelectrodes with varying Au NP densities were obtained by adjusting the sputtering duration. The obtained photoelectrodes are referred to as SiC/Au‐15, SiC/Au‐30, SiC/Au‐45, and SiC/Au‐60 photoelectrodes corresponding to the set sputtering duration of 15, 30, 45, and 60 s, respectively. Figure 1b–d shows the top‐view SEM images of the etched SiC nanostructures at different magnifications. The images clearly show that the etched SiC nanostructures had a large‐scale and highly oriented 1D NW configuration uniformly distributed throughout the etched area. Figure 1e shows a cross‐sectional view of the SiC nanoarrays. The etched depth of the NWs was ≈17.5 µm, and all the NWs were perpendicular to the SiC wafer surface with uninterrupted geometry, further demonstrating highly ordered NW arrays. Moreover, the well‐aligned NWs had uniform diameters of ≈26.89 nm with an aspect ratio reaching 650.80. The 1D NW arrays with high specific surface areas were beneficial for electrochemical reactions and the transmission and conversion of photoelectrons during PEC photodetection.^[^ ^23^ ^]^ Figure 1f,g presents the top‐view SEM images of the SiC/Au‐30 composite nanoarrays at different magnifications. The similar morphologies of the original SiC NW arrays (Figure 1b–d) and those of the SiC/Au‐30 nanoarrays suggested that surface decoration by the Au NPs via sputtering did not destroy the SiC nanoarrays geometry. Furthermore, numerous isolated Au NPs were uniformly decorated on the surface of the SiC nanoarrays rather than forming a continuous film (Figure S1, Supporting Information), which are expected to play a synergistic role in the optoelectronic application of the photoelectrodes.^[^ ^24^ ^]^ Figure S2a–c (Supporting Information) shows the elemental distribution mappings of C, Si, and Au within the SiC/Au composite nanoarrays. The mappings showed that these elements were distributed evenly, which further confirmed that the Au NPs had been deposited uniformly on the nanoarray surfaces.

Figure 1h presents a representative low‐magnification TEM image of an individual SiC/Au composite NW, showing that Au NPs with similar sizes are uniformly distributed on the surface of the SiC NW. Based on statistics from more than 30 NPs, the average diameter of the Au NPs is estimated to be ≈5.91 nm with an error of 0.08 nm. Figure 1i displays a high‐resolution transmission electron microscopy (HRTEM) image of a partial region of the composite NW. The measured interplanar spacing between two adjacent lattice fringes of the exposed SiC NW is ≈0.239 nm, corresponding to the (102) crystal plane of 4H‐SiC. Simultaneously, the lattice fringes of the NPs in the image show an interplanar spacing of ≈0.235 nm, which matches the (111) plane of cubic Au. The information presented in the image clearly shows that the Au NPs are firmly dispersed on the exterior of the SiC NP, providing additional evidence for the formation of the SiC/Au heterojunction. In addition, the selected‐area electron diffraction (SAED) pattern of the SiC/Au NW exhibits two characteristic diffraction rings consistent with the (111) and (311) planes of cubic Au, as shown in Figure 1j. Furthermore, the element distribution of a single nanowire is also shown in Figure S2d–f (Supporting Information). It is noteworthy that the Au element is concentrated and distributed exclusively at the nanoparticle regions with almost no distribution detected in other areas, as shown in Figure S2f (Supporting Information), which further confirms that the Au exists on the SiC NW surface in the form of nanoparticles rather than as a continuous film.

The XRD patterns of the original SiC nanoarrays and SiC/Au‐30 nanoarrays were recorded, as shown in Figure S3a (Supporting Information). These patterns revealed that the SiC nanoarrays with and without Au NPs decoration comprised the pure 4H‐SiC phase (JPCD card no. 29–1127), indicating that the ion‐sputtering process did not affect the phase composition of the original SiC nanoarrays. The absence of an Au signal in the XRD pattern of the Au‐NP‐modified nanoarrays may be due to the low Au content.^[^ ^25^ ^]^ Figure S3b (Supporting Information) shows the Raman spectra of the original SiC and SiC/Au‐30 nanoarrays samples. Five peaks corresponding to the longitudinal acoustic (LA) mode E_2_ (203.16 cm^−1^), LA mode A_1_ (610.03 cm^−1^), transverse optical (TO) mode E_2_ (776.77 cm^−1^), TO mode E_1_ (TO, 798.27 cm^−1^), and longitudinal optical (LO) mode A_1_ (977.05 cm^−1^) were detected in the Raman spectra of the original SiC nanoarrays.^[^ ^21^ ^]^ The Raman peaks of the composite sample were more intense than those of the original SiC nanoarrays. This difference was mainly attributed to the high proportion surface atoms of the attached small‐sized Au NPs, which strengthened the Raman signal of surrounding molecules via LSPR (the surface‐enhanced Raman scattering effect).^[^ ^26^ ^]^ The Raman peak of Au was not observed, mainly due to the low content of Au.^[^ ^27^ ^]^ The chemical composition and elemental bonding of the SiC/Au‐30 sample were analyzed by XPS (Figure S3c, Supporting Information), confirming that the dominant elements in the sample were Au, Si, C, O, and F. The F element mainly originated from the residue of the HF solution on the nanoarray surfaces.^[^ ^21^ ^]^ Three splitting peaks of Si 2p located at 98.77, 101.96, and 104.55 eV were detected (Figure S3d, Supporting Information), corresponding to Si─Si, Si─C, and Si─O bonds, respectively.^[^ ^28^, ^29^ ^]^ The existence of the Si─Si bond was attributed to the fact that the C sites in some regions of SiC were not occupied.^[^ ^30^ ^]^ Figure S3e (Supporting Information) shows three peaks of C 1s, which correspond to the C─Si bond (283.94 eV), C─C bond (284.53 eV), and C─H bond (285.48 eV).^[^ ^31^ ^]^ The C─H bond originated from hydrocarbons adsorbed from the environment.^[^ ^32^ ^]^ Figure S3f (Supporting Information) shows the binding peaks of Au 4f, revealing two splitting peaks centered at 84.71 and 88.39 eV, corresponding to Au 4f_7/2_ and Au 4f_5/2_, respectively.^[^ ^33^ ^]^ These results further verified the successful fabrication of the SiC/Au composite structure.

Figure S3g (Supporting Information) shows the PL spectra of the SiC and SiC/Au‐30 samples. The spectra of both samples show clear emission peaks at 616 nm attributed to the defect level inside the SiC.^[^ ^34^ ^]^ Impressively, PL quenching occurred in SiC/Au‐30 after Au NP deposition. The result indicates that a heterojunction successfully formed between the SiC nanoarrays and the Au NPs, leading to the effective separation of the photogenerated electron–hole pairs. This separation imparts excellent advantages in the photodetection applications of the developed device.^[^ ^20^ ^]^ In addition, time‐resolved photoluminescence (TRPL) spectroscopy was performed on the two samples, as shown in Figure S3h (Supporting Information). The double exponential decay equation was employed, and the corresponding fitting parameters are listed in Table S2 (Supporting Information).^[^ ^1^, ^35^ ^]^ The result reveals that the Au‐modified SiC nanoarrays undergo a rapid decay process with the carrier lifetime decreasing from 2.35 to 1.75 ns, further proving that the Au NPs are crucial for accelerating the carrier separation and improving the carrier migration and transfer efficiency within the SiC/Au‐30 photoelectrode.^[^ ^36^ ^]^ In addition, the absorption spectra of the SiC and SiC/Au heterojunction nanoarrays were measured, as depicted in Figure S3i (Supporting Information). The nanoarrays exhibit pronounced light absorption in the UV region before and after forming the composite device, and the absorption edge is located at ≈400 nm, revealing its wide bandgap characteristics and suitability for UV‐light detection applications.

**Figure 2:**
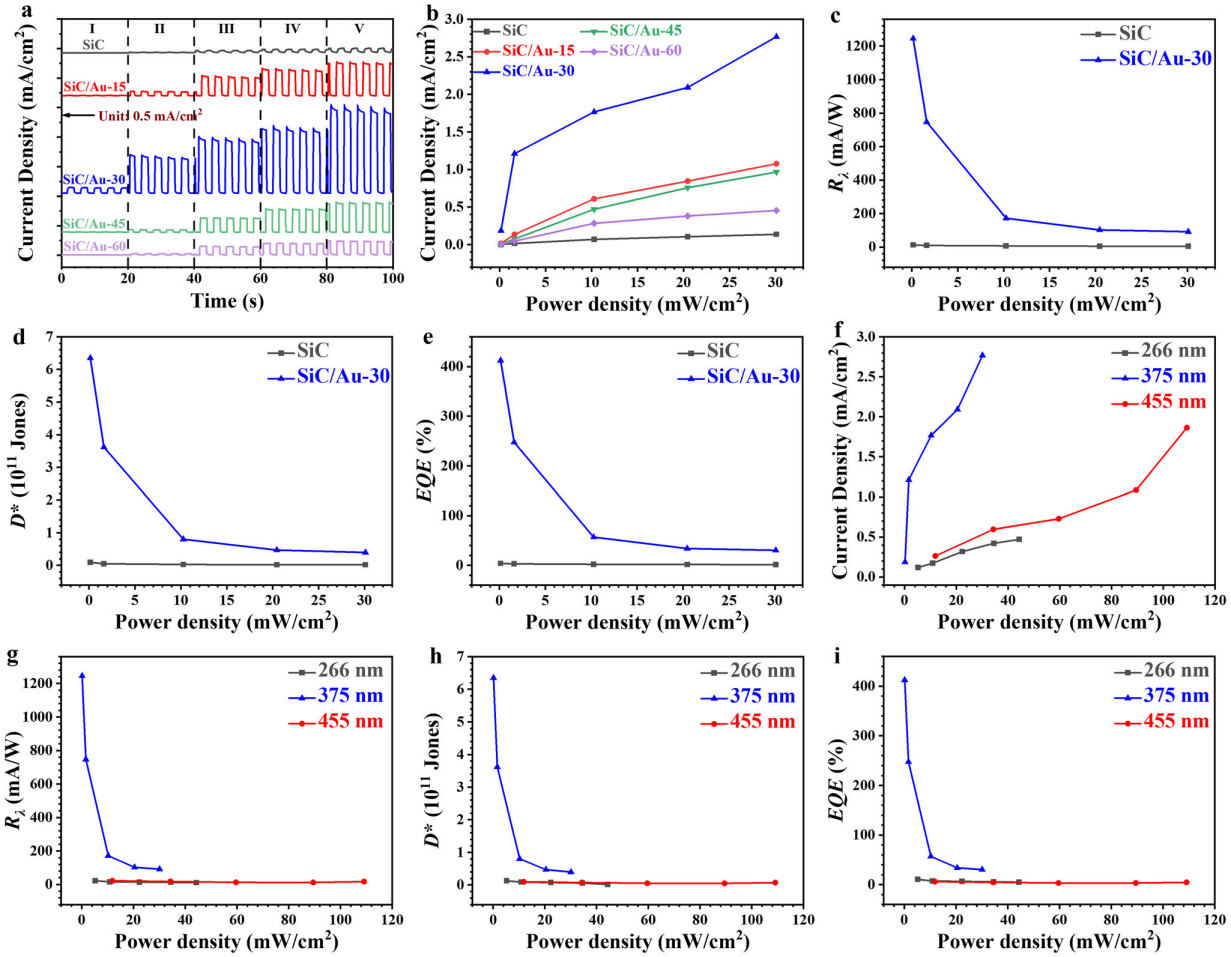
The photodetection performance of original SiC nanoarrays and SiC/Au heterojunction nanoarrays in 0.5 M KOH solution with an applied bias of 0.6 V and 375 nm UV light illumination under different optical power densities: a) Transient photocurrents, b) photocurrent density, c) responsivity (*R_λ_*), d) detectivity (D^*^), e) external quantum efficiency (*EQE*), respectively. The photodetection performance of SiC/Au‐30 nanoarrays in 0.5 m KOH aquatic solution under an applied bias of 0.6 V and illumination at wavelengths of 266, 375, and 455 nm. f) Photocurrent density, g) responsivity (*R_λ_*), h) detectivity (D^*^), i) external quantum efficiency (*EQE*), respectively.

To systematically investigate the influence of the deposited Au NPs on the photodetection performance of the SiC nanoarrays, PEC UV PDs based on original SiC nanoarrays and those based on the SiC/Au heterojunction nanoarrays with different sputtering durations (15, 30, 45, and 60 s) were constructed. The photodetection testing of the PEC PDs was conducted in a 0.5 m KOH solution under 375 nm UV‐light irradiation with a bias voltage of 0.6 V. The transient photocurrent density curves of SiC and various SiC/Au nanoarrays under different optical power densities (Table S1, Supporting Information) were first recorded, as shown in **Figure** **2**a. The significant photocurrent fluctuations with two distinct states—i.e., when light illumination was set on and off—demonstrated excellent responsiveness and reproducibility to light switching of the constructed PEC PDs. Moreover, the photocurrent density of the PEC PDs gradually increased with increasing light intensity, revealing their high sensitivity to light intensity. The increase in the photocurrent density under high light intensity was attributed to the multiphoton absorption effect in the photoelectrode: multiple photons are simultaneously absorbed, resulting in a rapid increase in the number of excited‐state electrons.^[^ ^28^ ^]^ Figure 2b shows the corresponding photocurrent density curves of these five samples, clearly demonstrating that all the Au NP‐modified SiC nanoarrays have higher photocurrent density than the original SiC nanoarrays. Compared with the original SiC nanoarrays, the photocurrent density of the SiC/Au heterojunction nanoarrays increased significantly even after a 15 s sputtering duration, revealing the high sensitivity of the PEC PDs to the Au NP modification mainly because of the surface plasmon effect induced by the Au NPs. When the sputtering duration is extended to 30 s, the SiC/Au‐30 sample reaches the highest photocurrent density value: this observation shows that the surface plasmon resonance effect of the Au NPs is fully excited under the UV‐light illumination at this state. A large number of free electrons oscillate collectively, and the generated strong local electromagnetic field greatly enhances the light‐trapping ability, enabling more photons to be converted into photogenerated carriers. The photogenerated electrons can be efficiently transferred from the conduction band of SiC to the surface of the Au NPs. Furthermore, the distribution density of the Au NPs at this time ensures the smoothness of the electron transfer path, effectively reducing the recombination probability of the electron–hole pairs and further improving photocurrent generation efficiency. In addition, the increased specific surface area of the Au NPs provides abundant active sites for the PEC reaction, allowing more reactants to be adsorbed on the surface of the photoactive material; this indirectly promotes the increase of the photocurrent density as well.^[^ ^37^ ^]^ However, when the sputtering duration was further extended to 45 s and 60 s, the photocurrent density showed a clear downward trend. This might have been due to the agglomeration of excessive Au NPs on the surface of the SiC nanoarrays, covering some active sites and thus decelerating light absorption and carrier transport. Moreover, the agglomerated Au NPs might also have weakened the LSPR effect and formed local regions unfavorable for electron transfer, increasing the recombination centers of the electron–hole pairs and thereby reducing the photocurrent density. These results show that the Au NP density plays a crucial role in determining the photodetection performance of the SiC/Au heterojunction nanoarrays. The SiC/Au‐30 sample achieved the highest photocurrent density through synergistic effects of ample active sites, optimized light absorption, and efficient carrier transport and separation.

In general, the photosensitivity of PEC PDs can be defined using *R*_λ_, *D* ^*^, and *EQE*. *R*_λ_ reflects the responsivity of a PD to optical signals. *D* ^*^ reflects the detection accuracy of weak signals in a noisy environment. *EQE* is the ratio of the number of carriers to the total number of absorbed photons and reflects the actual efficiency of the detector in converting incident photons into photogenerated carriers.^[^ ^21^ ^]^ To further assess the performance of the SiC NW arrays and the SiC/Au‐30 heterojunction nanoarrays, these three key parameters were calculated using the following formulas^[^ ^38^, ^39^ ^]^:

(1) $$R_{\lambda }=\frac{I_{ph}}{PA}=\frac{I_{light}-I_{dark}}{PA}\;$$

(2) $$D^{*}=\frac{R_{\lambda }}{\sqrt{2e\frac{I_{dark}}{A}}}\;$$

(3) $$EQE=\frac{hc}{e\lambda }\;R_{\lambda }$$

where *I*_dark_ is the current measured in darkness; *P* is the incident light power density; *A* is the effective area under light illumination, measured to be 0.5 cm^2^ in this study; *c* is the speed of light; *e* is the fundamental elementary charge; *h* is Planck's constant; and *λ* is the excitation wavelength.^[^ ^40^ ^]^ The *R*_λ_, *D* ^*^, and *EQE* values, which depend on the power density of light, were calculated and plotted in Figures 2c–e. The plots clearly demonstrate that these parameter values of the SiC/Au‐30 are significantly higher than those of the original SiC nanoarrays. This result further indicates that the construction of the heterojunction between the SiC and Au NPs remarkably enhanced the photosensitivity of the resulting material. Under the condition of a minimum light intensity of 0.15 mW cm^−2^, *R*_𝜆_, *D* ^*^, and *EQE* of SiC/Au‐30 reached their maximum values of 1244.95 mA W^−1^, 6.35 × 10^11^ Jones, and 412.45%, respectively, indicating that SiC/Au‐30 has great potential in detecting weak UV signals in underwater environments. Notably, the *EQE* of the fabricated SiC/Au‐30 sample was the highest among typical semiconductor‐nanostructure‐based PEC PDs reported to date (**Table** **1**). The other parameters also meet the state‐of‐the‐art requirements.

**Table 1:** Key parameters of typical PEC PDs reported in the literatures.

PEC PDs	Wavelength [nm]	Solution	Applied bias [V]	*R_λ_* [mA/W]	*EQE* [%]	D^*^ [Jones]	Rise/Decay times [ms]	Refs.
*a*‐Ga_2_O_3_/CFP	254	Na_2_SO_4_	0	12.9	6.3	–	150/130	[10]
Rh–Cr_2_O_3_ / AlGaN	255	H_2_SO_4_	0	178.3	86.7	‐	53/55	[20]
2D InSe	365	KOH	0	10.14	34.5	3.68 × 10^9^	2/37	[43]
GaN/CsPbBr_3_	310	Water	0	1.08	0.43	–	740/7200	[44]
α‐Ga_2_O_3_@a‐Al_2_O_3_	254	Na_2_SO_4_	0	22.7	–	2.2 × 10^11^	–	[45]
Cu@GaN	458	Seawater	0	5.04	–	–	0.68/1.43	[46]
SiC NWs	375	Na_2_SO_4_	0.6	218.77	72.47	6.64 × 10^13^	17/48	[47]
GeH	365–730	Na_2_SO_4_	0	0.023	–	6.7 × 10^7^	240/740	[48]
InP/ZnSeS	600	KOH	0	664	–	2.8 × 10^11^	–	[49]
SiC/SnO_2_	375	Seawater	0	1.97	65.5	1.98 × 10^11^	30/120	[28]
GaSe	455	H_2_SO_4_	−0.3	160	–	–	855/720	[50]
Quintuple heterotypic homojunction GaAs	850	Na_2_SO_3_	0	20.4	–	1.46 × 10^12^	1.1/1.1	[51]
Cr_2_S_3_	350	KOH	0.6	0.005354		4.28 × 10^7^	60/60	[52]
GaN/Co_3_O_4_	340	PBS	0	217.2	79.2	–	0.03/0.03	[53]
Graphene quantum Dots	365	KCl	0.6	0.01241	–	–	260/1500	[19]
CuO	455	Seawater	0	−83.18	–	−9.34±0.5 × 10^10^	28/140	[54]
PbTe	Simulated light	KOH	0.6	0.0831	–	–	900/‐	[55]
In_2_O_3_	365	KOH	0	44.43	–	8.55 × 10^10^	20/30	[56]
AlGaN/Pt–GaN	254	H_2_SO_4_	0	3.7	–	–	–	[57]
Pt/GaN	365	H_2_SO_4_	0	42.4	–	–	–	[11]
Violet phosphorus	350	KOH	0	0.0317	–	7.92 × 10^10^		[58]
SiC/Au‐30	375	KOH	0	108.19	35.84	1.35 × 10^11^	170.19/230.71	This work
		KOH	0.6	1244.95	412.45	6.35 × 10^11^	86.5/170.95	
		H_2_SO_4_	0.6	60.03	19.89	–	–	
		Na_2_SO_4_	0.6	909.32	301.26	–	–	

The superior photosensitivity of the SiC/Au‐30 PEC UV PD constructed in current work was dependent on not only the promoting effect of the Au NPs with the optimal density but also the following aspects: 1) The highly oriented and well‐aligned 1D SiC NW arrays with uninterrupted geometry and a high aspect ratio enhanced the light‐harvesting capability of the resulting material and allowed multiple refractions of light within the NW array forest, resulting in improved light absorption. Moreover, the well‐aligned NWs had a large surface with numerous redox active sites, facilitating interactions with incident light and the electrolyte and accelerating the separation and transport of photogenerated charge carriers. 2) The built‐in electric field within the established heterojunction affected band bending at the interface and offered a driving force for separating photoexcited carriers. When subjected to above‐bandgap UV‐light illumination, the photoexcited electron–hole pairs were rapidly separated by the built‐in electric field existing in the depletion region. This increased the sensitivity of the SiC/Au‐30 PEC PD. 3) The photoelectrode had an integrated self‐supporting geometry. Traditional PEC PDs are formed by assembling heterojunction nanostructures by techniques such as spin coating and physical or chemical vapor deposition.^[^ ^4^ ^]^ These methods cannot ensure that all the carrier transport channels of inner nanostructures are effectively and firmly connected to the current collector. Meanwhile, in the proposed PEC PD, the inner SiC NWs were firmly connected with the current collector without any interfaces, resulting in significantly enhanced carrier transport and photodetection performance. 4) The incorporation of Au NPs with SiC NWs to form a composite structure not only modifies the original interface architecture but also introduces the LSPR effect: i) The attached Au NPs can introduce interface defects at their junction with the SiC NWs. The presence of these defects disrupts the periodic potential field generated by the strictly periodically arranged atoms. Thus, energy levels that permit electron access into the forbidden band of the semiconductor are formed, which can freely exchange electrons with the conduction band, acting as traps. Due to the trapping of holes at the interface, these traps subsequently generate photoconductive gain.^[^ ^41^ ^]^ ii) The enhanced PEC performance of the SiC/Au photoelectrode can be partially attributed to the electric field amplification induced by the LSPR effect. The electromagnetic field around the Au NPs is intensified due to plasmonic resonance,^[^ ^33^ ^]^ which subsequently enhances the photon absorption capacity of the adjacent SiC. It is noteworthy that the LSPR effect of Au NPs can transfer energy to the SiC nanoarray not only through radiative processes (such as scattering and electric field enhancement) but also via non‐radiative processes, including hot electron injection and plasmon resonance energy transfer.^[^ ^42^ ^]^ Consequently, the loaded Au NPs can also significantly promote carrier transport on the surface of SiC NWs.

To further explore the spectral response characteristics of the fabricated SiC/Au PEC PDs, their photoresponses under light illumination (266, 375, and 455 nm) with different optical power densities were systematically investigated. First, the transient photocurrent density of the original SiC and SiC/Au nanoarrays under a bias voltage of 0.6 V and in a 0.5 m KOH solution was measured (Figure S4, Supporting Information). The SiC/Au‐30 PEC PD exhibited optimal photoresponse behavior under light with different wavelengths. This revealed the remarkable ability of the PD to generate electrical signals under different light conditions and fully reflected its excellent broad‐spectral‐response characteristics. Moreover, the PEC PDs generated the highest photocurrent under 375‐nm light illumination, as shown in Figure 2f. Figure 2g–i shows the corresponding *R*_λ_, *D* ^*^, and *EQE* values of the PEC PD for light with different wavelengths. The *R*_λ_, *D* ^*^, and *EQE* values of SiC/Au‐30 photoelectrode at 375‐nm illumination were significantly higher than those at other wavelengths, indicating that the SiC/Au‐30 heterojunction nanoarrays converted incident light into electrical signals more effectively under 375‐nm light than under light with other wavelengths. This result further confirmed that despite its broad spectral responsiveness, the SiC/Au‐30 PEC PD achieved a nearly perfect synergy of light absorption and carrier generation, transport, and separation under 375‐nm light, greatly improving the utilization efficiency of photons and fully demonstrating its significant advantages as a high‐performance PEC PD.

**Figure 3:**
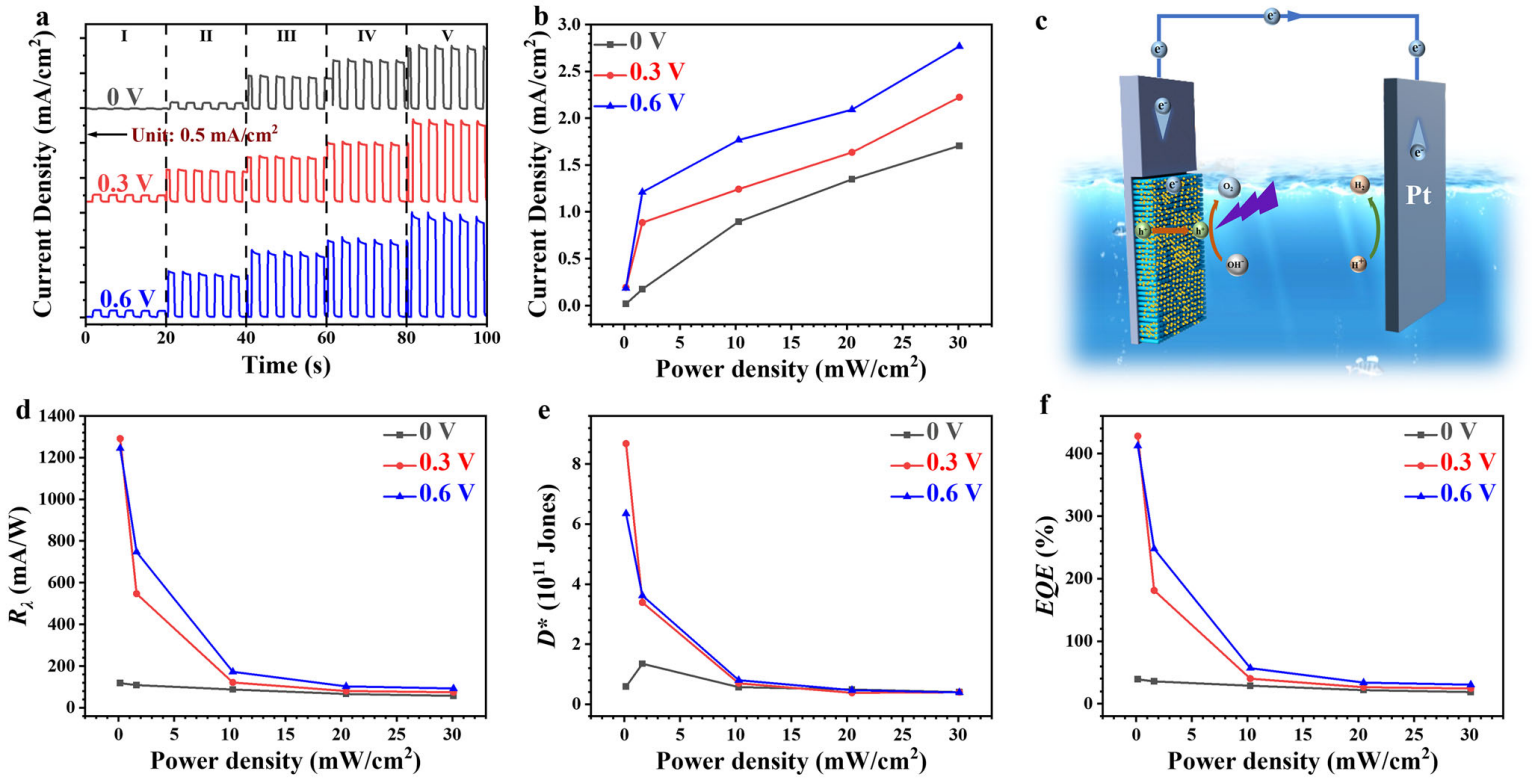
a) Transient photocurrent and b) photocurrent curves of the SiC/Au‐30 PEC PD under 375‐nm UV‐light illumination with applied biases of 0, 0.3, and 0.6 V and different optical power densities. c) Schematic illustrating the self‐powered operation mechanism of the SiC/Au PEC PD. d) *R*_λ_, e) *D* ^*^, and f) *EQE* values of the SiC/Au‐30 PEC PD.

The photosensitivity of the SiC/Au‐30 PEC PD under different bias voltages (0, 0.3, and 0.6 V) was also investigated under 375‐nm UV‐light irradiation with different optical power densities, as shown in the transient photocurrent density curves in **Figure** **3**a. The sharp increase and decrease in the photocurrent directly reflect the favorable response characteristics of the PEC PD to the turning on and off of light illumination. Generally, the response time quantitatively characterizes the transient response behaviors of the PEC PDs.^[^ ^59^ ^]^ The response time curves of the original SiC nanoarrays and SiC/Au‐30 nanoarrays PEC PD with the bias voltage of 0.6 V are shown in Figure S5 (Supporting Information). These curves show that, compared with the original SiC nanoarrays, the *τ*_r_ and *τ*_d_ of the SiC/Au‐30 heterojunction nanoarrays are decreased from 632.6 to 86.5 ms and from 988 to 170.95 ms, respectively. The reduction in the response times demonstrates the outstanding transient response performance of the SiC/Au‐30 PEC PD. Moreover, the SiC/Au‐30 nanoarrays also present rapid *τ*_r_ and *τ*_d_ of 170.19 and 230.71 ms even under the self‐powered condition (Figure S6, Supporting Information), which are within the state‐of‐the‐art range for semiconductor‐based PEC PDs reported thus far, as listed in Table 1. The origin of this elevated photoresponse mainly lies in the heterojunction structure formed by the SiC and Au NPs, which optimizes the electron transport path of the PEC PD. The Au NPs uniformly distributed on the surface of the SiC nanoarrays form an efficient “electron highway”, which greatly reduces the transport distance and time of the photogenerated carriers.^[^ ^60^ ^]^ The photogenerated electrons can be quickly transferred from the conduction band of SiC to the surface of the Au NPs through the Au NP channels under light illumination, rapidly generating a photocurrent in the external circuit and significantly shortening the response time.

In addition, the photocurrent density shows a remarkably upward trend as the bias voltage gradually increases from 0 to 0.6 V, as shown in Figure 3b. The evaluated photocurrent density could also be attributed to the enhancement of the electromagnetic field near the Au NPs by the unique plasmon resonance effect. The amplification of the electromagnetic field subsequently enhances the photon absorption capacity of the adjacent semiconductors.^[^ ^61^ ^]^ Moreover, since the electron–hole pair generation rate is proportional to the square of the local electric field intensity, an increase in the local electromagnetic field leads to the generation of the electron–hole pairs.^[^ ^62^ ^]^

Self‐powered PEC PDs, in which a built‐in electric field drives a self‐powered light detection mechanism, have been intensively studied. They have become the preferable choice of many applications owing to their compactness, simple fabrication steps, and ability to function independently and without an external power supply (which reduces energy consumption).^[^ ^43^, ^48^ ^]^ Specifically, in the current work, even in the self‐powered condition, the SiC/Au‐30 PEC PD exhibits a considerable photocurrent owing to its unique structure and outstanding photoelectric properties. Based on the difference between the energy levels of SiC and Au NPs, a built‐in electric field is spontaneously generated under light illumination. This electric field effectively promotes the separation and directional movement of photogenerated carriers, thereby generating a considerable photocurrent, as shown in the schematic diagram in Figure 3c. This self‐powered nature of the SiC/Au‐based PEC PD endows it with the capacity to accurately detect and rapidly respond to optical signals without an external power supply, greatly expanding its application potential in various scenarios, such as complex underwater monitoring and microsensor networks, lacking access to an external power supply.

Figure 3d–f shows the calculated *R*_λ_, *D* ^*^, and *EQE* values under different bias voltages. The SiC/Au‐30 PEC PD was exceedingly photosensitive even in an environment with a low optical power density—this property is crucial for application scenarios with extremely high requirements for sensitivity and efficiency, such as nighttime monitoring in low‐light‐level environments and deep‐sea optical signal detection. Notably, even in the self‐powered mode, the *R*_λ_, *D* ^*^, and *EQE* of the SiC/Au‐30 PEC PD reached up to 108.19 mA W^−1^, 1.35 × 10^11^ Jones, and 35.84% under the lowest optical power density of 1.62 mW cm^−2^, represent considerable advantages over the typical self‐powered PEC UV PDs reported to date (Table 1), indicating that the self‐powered PEC PD is extremely sensitive to the complex weak light environment. This implied that a multitude of incident photons can be efficiently converted into photogenerated carriers even in the absence of an external bias voltage. Thus, the excellent photoelectric conversion ability of the developed PEC PD in the self‐powered condition was demonstrated, laying a solid foundation for the wide application of this PD in the field of photodetection.

**Figure 4:**
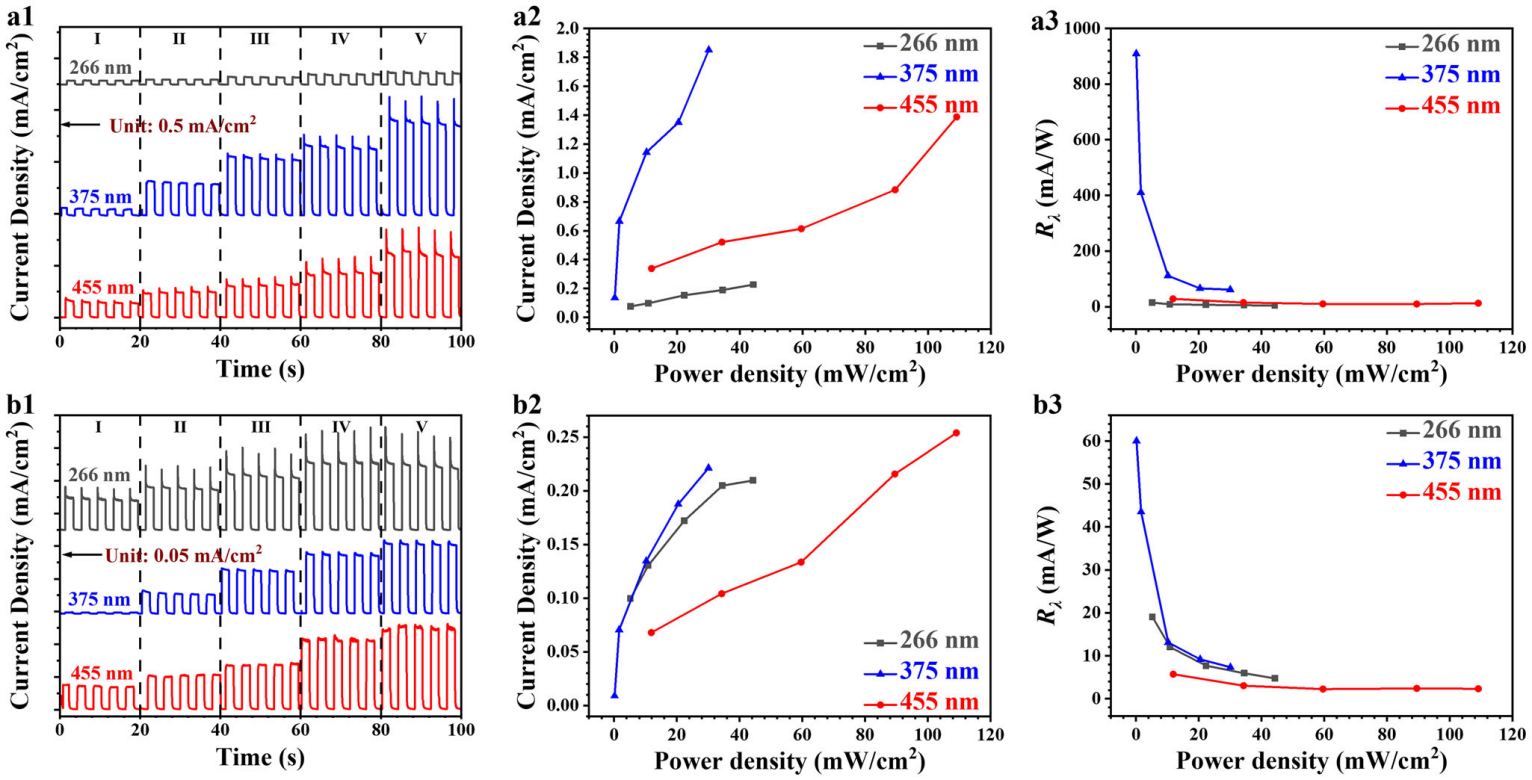
Photodetection performance of the SiC/Au‐30 PEC PD in a 0.5 m Na_2_SO_4_ solution under incident light with different wavelengths and power densities with an applied bias voltage of 0.6 V: a1) transient photocurrent curves, a2) photocurrent density, and a3) *R*_λ_. Photodetection performance of the SiC/Au‐30 PEC PD in a 0.5 m H_2_SO_4_ solution under incident light with different wavelengths and power densities with an applied bias voltage of 0.6 V: b1) transient photocurrent curves, b2) photocurrent density values, and b3) *R*_λ_.

Stable operation is a prerequisite for high‐performance pH‐universal adaptable PEC PDs for full aquatic optical communication. However, most reported PEC PDs face challenges in terms of the stability of their photoresponse in harsh acidic and alkaline environments. Acidic and alkaline conditions can cause severe corrosion and damage to the PD materials, causing severe deterioration of PD performance and service life, and even failure. To comprehensively examine the photoresponse properties of the SiC/Au‐30 PEC PD in harsh underwater environments, the photoresponse characteristics in 0.5 mol L^−1^ H_2_SO_4_ and 0.5 mol L^−1^ Na_2_SO_4_ solutions were systematically studied under incident light with different wavelengths and power densities. **Figure** **4**a1,b1 shows the transient photocurrent responses of the SiC/Au‐30 sample, confirming that the as‐constructed PEC PD showed excellent stability and reproducibility. The findings indicate that the SiC/Au‐30 PEC PD exhibited excellent photodetection performance in the 0.5 mol L^−1^ KOH electrolyte solution (Figures 2 and 3), and generated stable photoresponses in acidic and neutral environments. The ability of the developed SiC/Au‐30 PEC PD to adapt to complex and harsh operating environments and its wide pH adaptability are mainly attributed to the following aspects: 1) As a typical third‐generation wide‐bandgap semiconductor, SiC has a high chemical bond energy and outstanding chemical stability, making it highly resistant to chemical reactions in acidic and alkaline environments. 2) The Au NPs are extremely inert and hardly undergo any chemical reactions in acidic and alkaline environments. Hence, they form a physical protective barrier for SiC. 3) The modification by the introduction of stable Au NPs has multiple synergistic effects. The SiC/Au heterojunction generates a special charge transfer and distribution mechanism at the interfaces. In acidic or alkaline solutions, the interfacial property helps stabilize the processes of generation and transport of the photogenerated carriers.^[^ ^63^ ^]^ The photogenerated carriers can be efficiently and stably separated and transported under the synergistic effect of the composite structure under light irradiation. 4) The photoactive layer (including SiC nanoarrays and surface‐modified Au NPs) and current collector (the unetched portion at the bottom of the SiC nanoarrays) of the SiC/Au photoelectrode are integrated into a robust and stable self‐supporting configuration. This configuration is maintained reliably with limited carrier recombination even under harsh underwater conditions.^[^ ^38^ ^]^

In addition, the maximum photocurrent densities and highest responsivities of the SiC/Au‐30 PEC PDs in different solutions, as shown in Figures 4a2,a3,b2,b3 and 2b,c are summarized in **Table** **2**. The photocurrent densities and responsivities generally increase with increasing pH values in H_2_SO_4_, Na_2_SO_4_, and KOH solutions. This phenomenon is correlated with the photodetection principle of PEC PDs: Photons absorbed at the surface of a photoactive layer excite electrons, causing their transition from the valence band to the conduction band, resulting in numerous photogenerated electron–hole pairs. These charge carriers migrate to the photoelectrode surface and participate in redox reactions with the solution reactants.^[^ ^64^ ^]^ Variations in the pH of the liquid environment greatly influence the charge state of the electrode surface and the activity of the solution‐phase reactants.^[^ ^65^ ^]^ At elevated pH values, the photoelectrode surface becomes more negatively charged because of the OH^−^ occupying surface sites. These negative charges attract positively charged species, such as metal ions and cationic reactants, thereby increasing the adsorption and dissociation of the positively charged species at the electrode–solution interface. In addition, high pH promotes reactant ionization, further elevating their effective concentration near the electrode surface.^[^ ^66^ ^]^ Finally, the pH values of different liquid environments also affect the redox capability of the electrode surface.^[^ ^43^ ^]^ The redox activity at the electrode surface is enhanced at high pH levels because the OH^−^ ions participate in these reactions, thereby promoting the reaction rates.

**Table 2:** Variations in the photodetection behavior of the SiC/Au nanoarrays under an applied bias voltage of 0.6 V and Au sputtering duration of 30 s under light with different wavelengths and pH conditions.

Wavelength [nm]		*I*_ph_ [µA]			*R*_λ_ [mA/W]	
	H_2_SO_4_	Na_2_SO_4_	KOH	H_2_SO_4_	Na_2_SO_4_	KOH
266	104.82	113.42	241.8	14.69	19.04	22.47
375	110.59	925.53	1390	60.03	909.3	1244.95
455	126.99	693.56	948.3	5.73	28.57	22.115

To further investigate the dependence of the photoresponses of the SiC/Au‐30 nanoarray photoelectrode on the used solutions, EIS tests were performed in solutions with different pH values to understand the charge transfer process occurring on the photoelectrode surface (Figure S7, Supporting Information). The electrode–solution interface characteristics, the kinetic behavior of electron and ion transfer, and electrode reactions in the PEC PDs were examined by using Nyquist diagrams.^[^ ^67^ ^]^ The semicircle diameter and value in the Nyquist diagram represent the charge transfer resistance and number of interfaces, respectively.^[^ ^68^ ^]^ The semicircle diameter of the Nyquist diagram in the KOH solution was smaller than that in the other two solutions, indicating the low interface charge resistance in the KOH solution. This phenomenon not only improved the light response of the device but also reduced the accumulation of photogenerated carriers on the photoelectrode surface.

**Figure 5:**
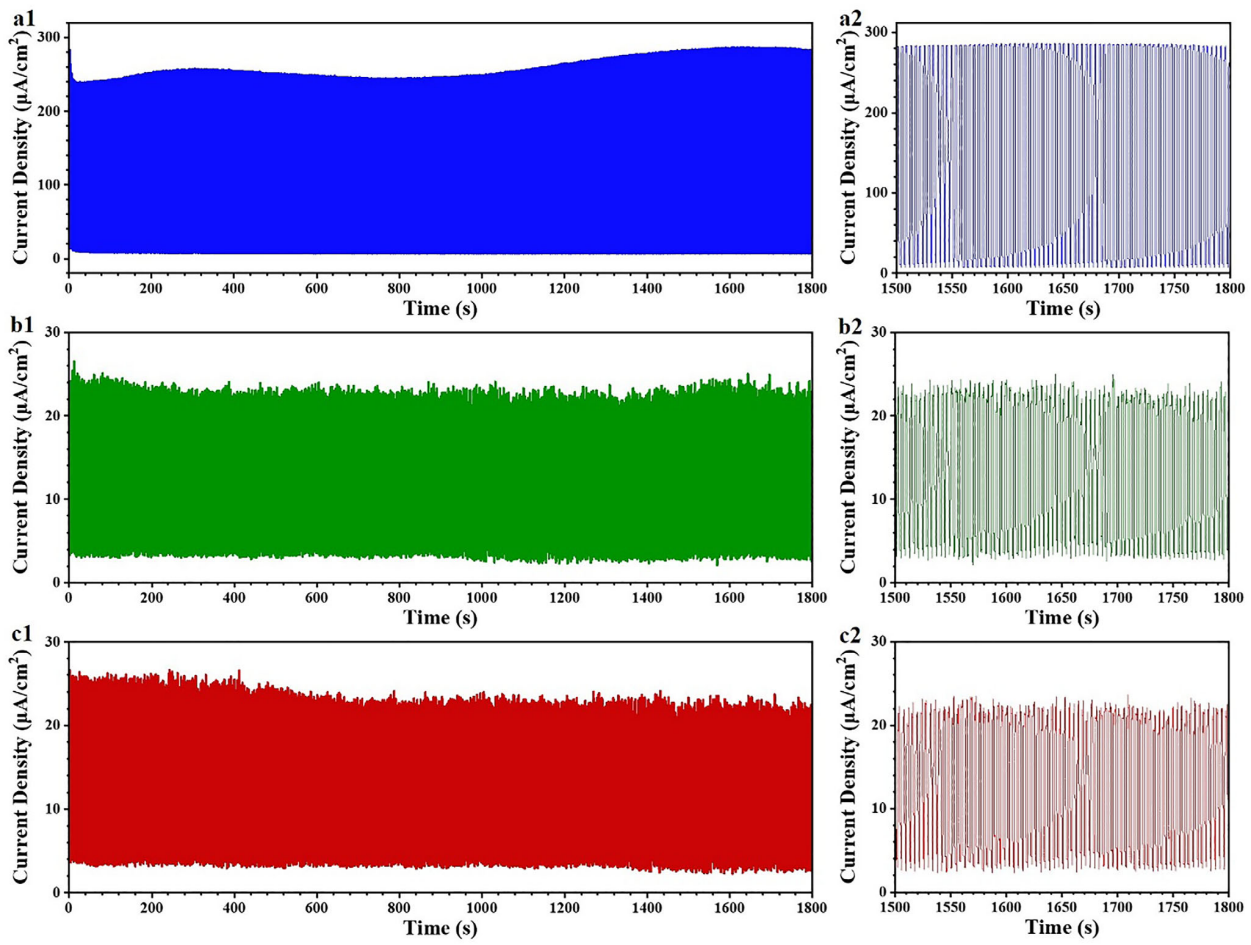
Long‐term stability evaluation of the SiC/Au‐30 PEC UV PD under 0.6 V after 1800 s of operation in different solutions: a1,a2) 0.5 m KOH, b1,b2) 0.5 m Na_2_SO_4_, and c1,c2) 0.5 M H_2_SO_4_.

Long‐term stability is important for evaluating the performance of PEC PDs in harsh full aquatic environments. This property ensures that devices can operate stably and reliably in practical applications, providing important evidence of their reliability and durability.^[^ ^38^ ^]^ The photocurrent–time responses of the developed SiC/Au‐30 PEC PD in solutions with different pH values under a bias voltage of 0.6 V and 375‐nm UV‐light illumination were monitored to systematically evaluate its long‐term stability. **Figure** **5**a1 shows the *I*–*t* curve in a 0.5 mol L^−1^ KOH solution over 1800 s; the device exhibited an initial photocurrent density of 282.2 µA cm^−2^. After 1800 s of light illumination, the photocurrent density was 280 µA cm^−2^ (a minimal decrease of 2.2 µA cm^−2^), confirming the excellent *I*–*t* response stability of the SiC/Au PEC PD. Additionally, the long‐term stability of the SiC/Au PEC PD in 0.5 mol L^−1^ Na_2_SO_4_ and 0.5 mol L^−1^ H_2_SO_4_ solutions was further investigated, as shown in Figure 5b1,c1, respectively. Similar to the situation in the KOH solution, the device demonstrated reliable stability in the Na_2_SO_4_ and H_2_SO_4_ solutions. Figure 5a2,b2,c2 shows the detailed on/off switching signals of the device in the three solutions over a duration of 1500–1800 s. Highly stable and reproducible transient photocurrent density curves were obtained with negligible fluctuations, further confirming the excellent photocurrent response stability of the PEC PD. In addition, the investigation about the surface morphology and composition of the SiC/Au nanoarrays before and after long‐term stability measurement was carried out based on the SEM and EDS technology, as shown in Figure S8 (Supporting Information). The results demonstrated that the surface structure of the SiC/Au nanoarrays presents similar morphology after long‐term tests in different electrolytes. Moreover, the main elements of C, Si, and Au are still evenly distributed, no new emerged impurity elements are detected. The exceptional long‐term stability under different pH values primarily originated from the synergistic effects of the inherent high chemical stability of SiC, robust integrated self‐supporting structure of SiC NWs, and their surface modified stable Au NPs.^[^ ^62^, ^69^ ^]^ The decorated Au NPs could greatly improve the stability and photo–corrosion resistance of the SiC/Au composite nanoarrays in the PEC reaction.^[^ ^70^ ^]^

**Figure 6:**
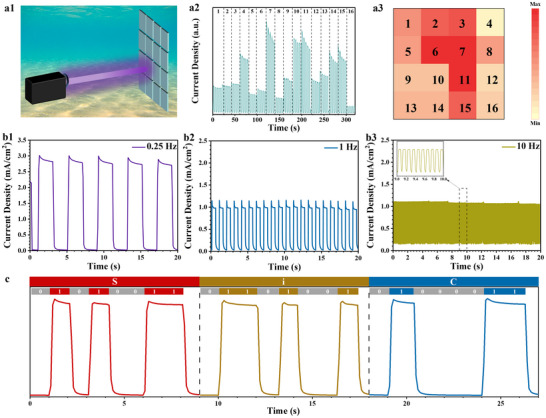
a1) Schematic of the underwater position detection application of the SiC/Au‐30 PEC PD. a2) Photoresponse curves for each sector in the 16‐sector partitioned device. a3) Photocurrent intensity mapping across the 16 sectors. b1–b3) Switching characteristics of the SiC/Au‐30 PEC PD at different frequencies. c) Signals after converting “S”, “i”, and “C” into ASCII codes.

Underwater imaging and position detection technology are becoming increasingly important for environmental monitoring and resource exploration. However, the development of advanced PDs for such applications faces major challenges, such as interference of environmental visible and infrared light, difficulty in device adaptability to harsh underwater environments, and high associated costs.^[^ ^22^ ^]^ The application of the SiC/Au‐30 PEC UV PD for underwater position detection was evaluated, as shown in the schematic in **Figure** **6**a1. The photoelectrode was divided into 16 sectors, and the *I*–*t* curves of each sector under UV‐light irradiation in a 0.5 m KOH solution were measured (Figure 6a2). The results demonstrated a non‐uniform photoresponse across different sectors of the photoelectrode, indicating that photon absorption at the photoactive material surface and subsequent electron–hole pair generation—thereby the photocurrent magnitude—were position dependent, thereby influencing the photocurrent magnitude. The photons were more readily absorbed by the photoactive materials when the illumination source approached the electrode, generating electron–hole pairs with a high density. The charge carriers migrated across the material surface and participated in enhanced redox reactions with solution‐phase reactants, ultimately generating strong photocurrent signals. Meanwhile, the photons had to traverse the thick catalyst and electrolyte layers to reach the electrode surface when the illumination source moved away from the photoelectrode, resulting in a reduced photon flux at the interface. This diminished photon availability reduced electron–hole pair generation and the photocurrent magnitude. As illustrated in Figure 6a3, each part of the photoelectrode analyzed a specific optical signal, encoding unique spatial information through distinct photocurrent signals, demonstrating the considerable application potential of the developed PEC PD for underwater positional mapping applications.

To validate the rapid response capability of the constructed PEC PD in practical underwater communication systems, the device was subjected to 375‐nm UV‐light illumination with shutter‐timing‐controlled switching frequency modulation. The photocurrent density progressively reduced with increasing switching frequency (Figure 6b1–b3). This result was mainly attributed to the reduced duration of illumination of the photoelectrode at high switching frequencies, which diminished the extent of redox reactions and consequently reduced charge carrier generation, resulting in low photocurrents.^[^ ^53^ ^]^ In addition, the device consistently exhibited distinct “on” and “off” states under 375‐nm light illumination across all tested frequencies, demonstrating its stable and rapid photoresponse and excellent reproducibility under complex practical conditions with frequency fluctuations. Finally, the string “SiC” was converted into a binary code based on the American Standard Code for Information Interchange (ASCII), where binary digits “0” and “1” corresponded to the dark and illuminated states, respectively, as shown in Figure 6c. The received current signals closely matched the original binary data, further demonstrating the promising underwater optical communication capabilities of the as‐constructed PEC PD.

## 3 Conclusion

An exceptional self‐powered PEC UV PD for robust underwater optical communication in full aquatic environments was established based on the integrated self‐supporting SiC/Au heterojunction nanoarrays. Modification using the Au NPs significantly enhanced the photoresponse performance of the SiC nanoarrays. Further, the SiC/Au heterojunction nanoarrays with a sputtering duration of 30 s exhibited optimal photodetection performance, with a rapid *τ*_r_/*τ*_d_, high *R*_λ_, remarkable *D* ^*^, remarkable *EQE* of 86.5/170.95 ms, 1244.95 mA W^−1^, 6.35 × 10^11^ Jones, and 412.45%, respectively, in a 0.5 m KOH solution under 375‐nm UV‐light illumination at a bias voltage of 0.6 V. The constructed SiC/Au‐30 PEC PD showed excellent self‐powering ability owing to the driving effect of the built‐in electric field formed by the SiC/Au heterojunction. In addition, the device also exhibited excellent long‐term stable photoresponse performance in acidic, neutral, and alkaline complex solutions. The excellent photodetection performance of the SiC/Au PEC PD, including the excellent wide‐pH adaptability, high long‐term stability, and self‐powered operation ability, was mainly attributed to the synergistic effects of the unique LSPR effect, built‐in electric field within the heterojunction, robust integrated self‐supporting photoelectrodes, highly oriented 1D nanoarrays benefits, and high chemical stability of SiC and Au NPs. These outstanding photoresponse characteristics in various complex solutions endow the constructed PEC UV PDs with considerable application potential in robust full aquatic optical communication, such as underwater environmental monitoring, ocean exploration, and biomedical detection.

## 4 Experimental Section

### Materials

Acetone (99%, C_3_H_6_O), ethanol (95%, C_2_H_6_O), and hydrofluoric acid (40%, HF) were purchased from Aladdin (Shanghai, China). Hydrogen peroxide (30%, H_2_O_2_), sodium sulfate (Na_2_SO_4_), sodium hydroxide (KOH), and sulfuric acid (H_2_SO_4_) were purchased from Sinopharm Chemical Reagent Co., Ltd. (Shanghai, China). Single‐crystal 4H‐SiC wafers (thickness = 350 µm) with a resistivity of ≈1 × 10^9^ Ω·cm were purchased from Shanxi SemiCore Crystals Co., Ltd. (Taiyuan, China). The SiC wafers were first cut into small sheets of 0.5 × 1.5 cm^2^. The small sheets were then placed in an ultrasonic cleaner and subjected to sequential ultrasonic cleaning with acetone, ethanol, and deionized water for 15 min. Subsequently, the small SiC sheets were immersed in a mixed solution of C_2_H_5_OH and HF at a volume ratio of 1:1 for 30 min to remove any residue. Finally, the SiC sheets were rinsed with deionized water, dried in a vacuum oven at 60 °C for 15 min, and then stored in centrifuge tubes filled with nitrogen.

### Preparation of Whole Single‐Crystal Integrated Self‐Supporting 4H‐SiC Nanoarray Photoelectrodes

An optimized two‐step anodization method was adopted to prepare 4H‐SiC NW arrays. A graphite cathode and a small SiC sheet anode were immersed in an electrolyte composed of C_2_H_5_OH, HF, and H_2_O_2_ with a volume ratio of 6:3:1. First, pulsed power (SOYI‐300‐5 M) with a cycle period of 0.8 ms (T_on_ = 0.4 ms and T_off_ = 0.4 ms) was applied at 17 V for 2 min. Subsequently, the applied voltage was increased to 30 V with a 100% duty cycle for ≈25 s to remove the formed cap layer. Finally, a second anodization process was conducted in the same electrolyte for 7 min to obtain a whole single‐crystalline integrated self‐supporting SiC NW array photoelectrode.

### Fabrication of Integrated Self‐supporting SiC/Au Heterojunction Nanoarray Photoelectrodes

The surface of the SiC nanoarrays was decorated with Au NPs by utilizing an ion‐sputtering apparatus. The sputtering durations were fixed at 15, 30, 45, and 60 s, and the resulting composite samples were labeled as SiC/Au‐15, SiC/Au‐30, SiC/Au‐45, and SiC/Au‐60, respectively. The obtained SiC/Au composite nanoarrays can be directly employed as the integrated self‐supporting photoelectrodes for PEC PDs.

### Characterization of the Obtained Samples

The morphology and elemental distribution of the resulting samples were investigated using field‐emission scanning electron microscope (SEM, S4800 Hitachi, Japan) equipped with an apparatus for energy‐dispersive X‐ray spectroscopy (EDS, Bruker, Germany), transmission electron microscopy (TEM, JEM‐F200 JEOL Ltd., Japan). For the structural and phase analyses, X‐ray diffraction (XRD) was conducted with a Cu K𝛼 radiation of wavelength 1.5406 Å (D8 Advance, Bruker, Germany). In addition, the molecular structure, chemical composition, and vibrational properties of the obtained samples were analyzed using a Raman microscope (InVia, Renishaw, Gloucestershire, UK). The surface chemical composition was investigated by X‐ray photoelectron spectroscopy (XPS, ESCALAB 250Xi, Thermo Fisher Scientific, USA). The UV–vis absorption spectrum was recorded using a Hitachi model U‐3310 spectrophotometer. The time‐steady photoluminescence (PL) spectra and time‐resolved PL spectra were recorded using a fluorescence spectrometer (FLS‐1000, Edinburgh Instruments, UK).

### Device Fabrication and Measurements

In a standard three‐electrode system, the photodetection behavior of the PEC UV PDs based on the integrated self‐supporting SiC/Au nanoarrays was systematically investigated using an electrochemical workstation (CHI660D, Shanghai Chenhua Instrument Co., Ltd., China). SiC/Au and Pt electrodes were employed as the working and counting electrodes, respectively. The reference electrode was an Ag/AgCl electrode (containing saturated KCl solution) in acidic and neutral solutions, and an Hg/HgO electrode (containing KOH solution) in an alkaline solution. Linear sweep voltammetry tests were conducted at a scan rate of 0.01 V s^−1^ within the range of −0.8 to 0.8 V. Electrochemical impedance spectroscopy (EIS) measurements were performed with an alternating current perturbation amplitude of 0.005 V across a frequency range of 1–10^5^ Hz. Current–time (*I*–*t*) curves were recorded under a fixed bias voltage of 0.6 V with a sampling interval of 0.02 s. In addition, the photodetection performance of the SiC/Au photoelectrode was systematically evaluated across five distinct power density levels (*I*–*V*, from low to high; see Table S1, Supporting Information) under different illumination wavelengths in 0.5 m KOH, 0.5 m Na_2_SO_4_, and 0.5 m H_2_SO_4_ electrolytes separately.

## Conflict of Interest

The authors declare no conflict of interest.

## Supporting information



Supporting Information

## Data Availability

The data that support the findings of this study are available from the corresponding author upon reasonable request.
