# Supplementary material for: Self‐Powered SiC‐Based Photoelectrochemical Ultraviolet Photodetectors for Robust Underwater Optical Communication Against Full Aquatic Environments
Source: Adv Sci (Weinh). 2025 Oct 13;13(2):e13939. doi: 10.1002/advs.202513939 (PMC12786366; doi:10.1002/advs.202513939)
Supplement: Supplementary file 1 — Supporting Information [file ADVS-13-e13939-s001.docx]

***Supporting Information***

**Self-powered SiC-based Photoelectrochemical Ultraviolet Photodetectors** **for Robust** **Underwater Optical Communication** **against** **Full** **Aquatic Environments**

*Runchao Dong^1,2,#^, Hulin Wang^1,2,#^, Jia Zhang^1^, Hongxin Yin^1^, Genqiang Liu^1^, Bartosz Orwat^3^, Beata Luszczynska^3^, Weijun Li^1^, Dongdong Zhang^1^, Huan He^2^, Zhentao Du^2^, Shanliang Chen^1,*^ and Weiyou Yang^1,*^*

*^1^ Institute of Micro/Nano Materials and Devices, Ningbo University of Technology, Ningbo, 315211, P.R. China*

*^2^ School of Resources, Environment, and Materials, Guangxi University, Nanning, 530004, P.R. China*

*^3^ Department of Molecular Physics, Faculty of Chemistry, Lodz University of Technology, Zeromskiego 116, Lodz 90–924, Poland*

***Corresponding Authors:***

Corresponding authors. E-mails: shanliangchen@nbut.edu.cn (S. Chen)

weiyouyang@tsinghua.org.cn (W. Yang)

# Equal contribution authors.

**Table S1.** The employed different optical power densities at different wavelengths in the photodetection measurement.

| **Power densities (*m*W/cm^2^)** | **I** | **II** | **III** | **IV** | **V** |
| --- | --- | --- | --- | --- | --- |
| **266 nm** | 5.23 | 10.87 | 22.34 | 34.5 | 44.3 |
| **375 nm** | 0.15 | 1.62 | 10.27 | 20.45 | 30.1 |
| **455 nm** | 11.85 | 34.3 | 59.6 | 89.5 | 109.1 |


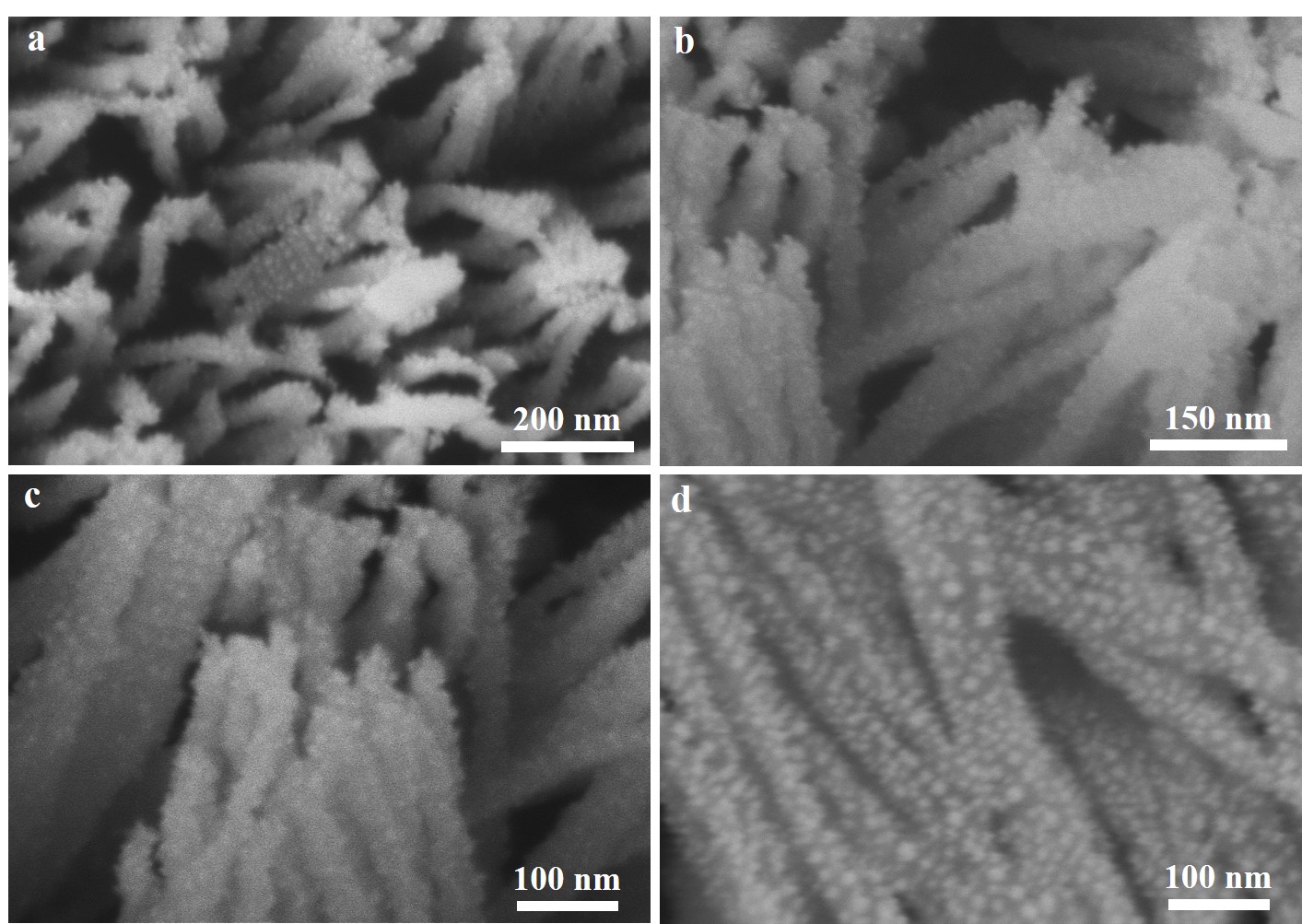


**Figure S1.** The top-view SEM images of SiC/Au heterojunction nanowire arrays under higher magnifications.

**
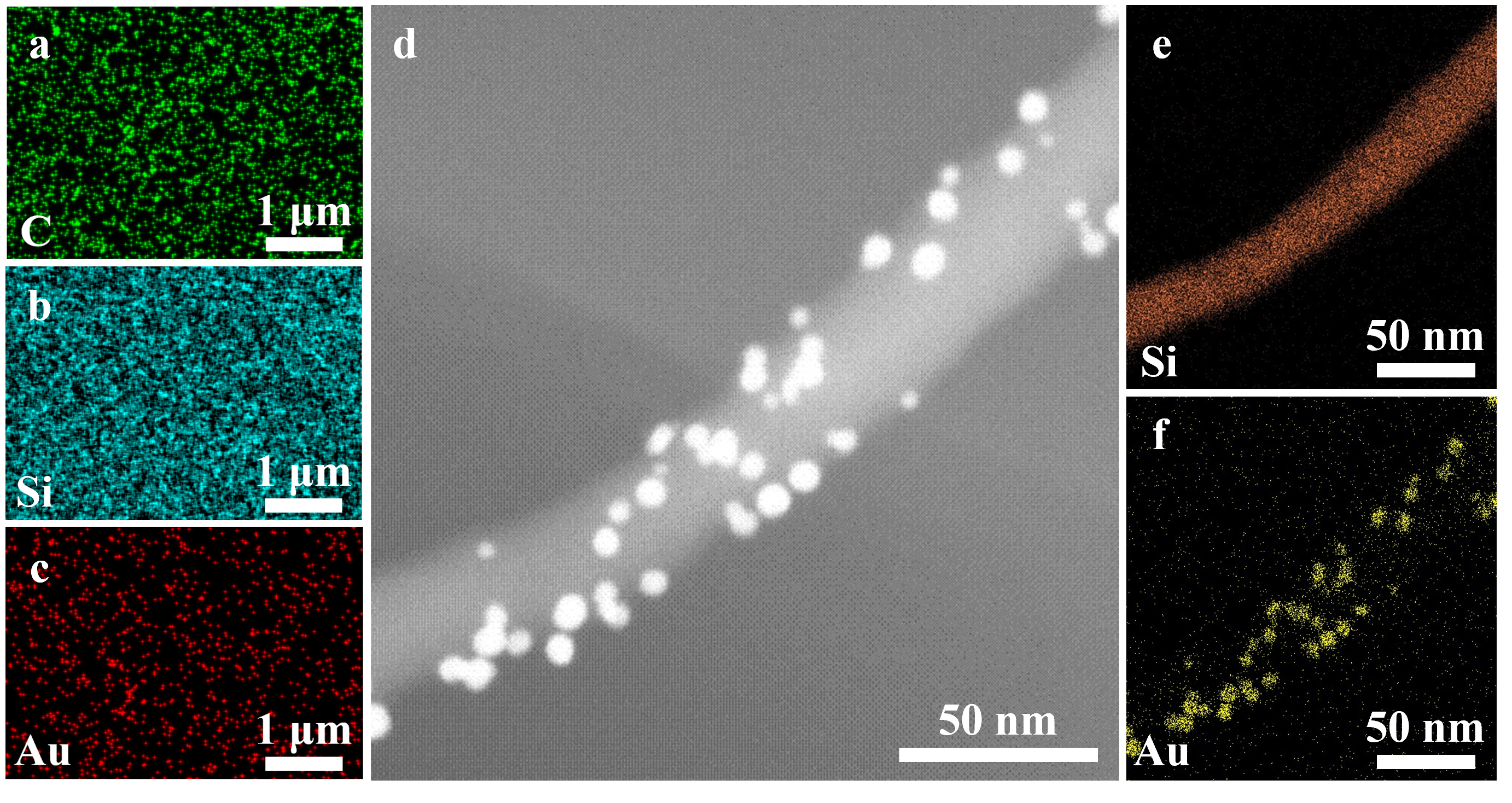
**

**Figure S2.** The corresponding elemental mappings of (a) C, (b) Si, and (c) Au within the SiC/Au composite nanoarrays, respectively, based on the SEM technique. (d) The typical TEM image of a SiC/Au composite nanowire. The corresponding elemental mappings of (e) Si, and (f) Au within the SiC/Au composite nanowire, respectively, based on the TEM technique.


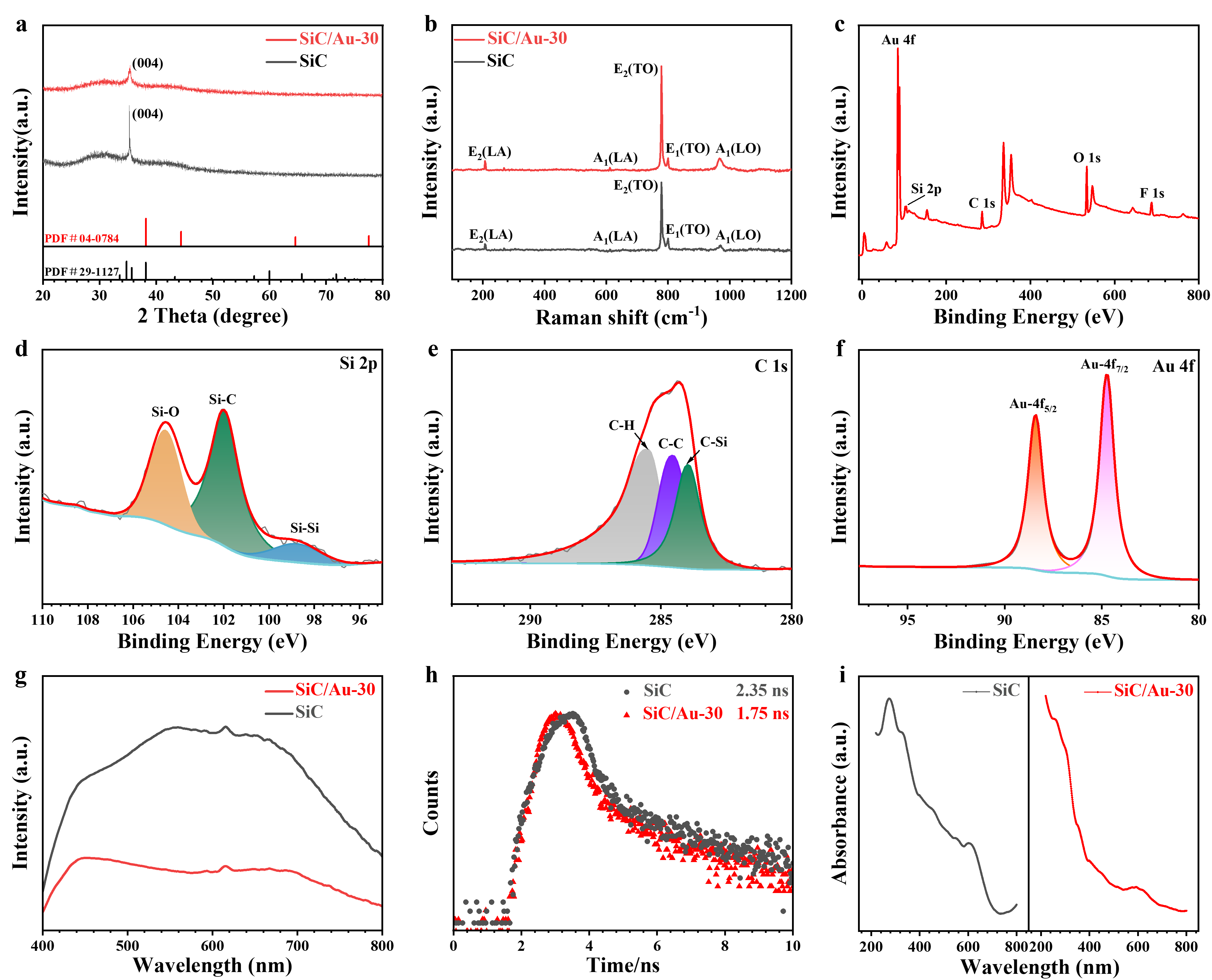


**Figure S3.** (a) XRD patterns and (b) Raman spectra of original SiC nanoarrays and SiC/Au-30 composite nanoarrays, respectively. (c) XPS spectrum of the SiC/Au-30 composite nanoarrays. (d-f) High-resolution XPS spectra of Si, C, and Au, respectively. (g) Room-temperature PL spectra, (h) time-resolved PL spectra, and (i) absorption spectra of the SiC nanoarrays and SiC/Au-30 composite nanoarrays, respectively.

**Table S2.** The parameters of the TRPL decay spectra fitted by a biexponential function.

| **Samples** | ***τ*_1_ (ns)** | ***τ*_2_ (ns)** | **A_1_ (%)** | **A_2_ (%)** | ***τ* (ns)** |
| --- | --- | --- | --- | --- | --- |
| **SiC Nanoarrays** | 0.3947 | 4.305 | 91.61 | 3.39 | 2.35 |
| **SiC/Au-30 Nanoarrays** | 0.5047 | 3.805 | 92.51 | 7.49 | 1.75 |

Note: I(t) = A_1_ exp(-t/τ_1_) + A_2_ exp(-t/τ_2_), where I(t) is the PL signal’s intensity, *τ*_1_ and *τ*_2_ are the decaying lifetimes, and A_1_ and A_2_ are their corresponding proportions.

**
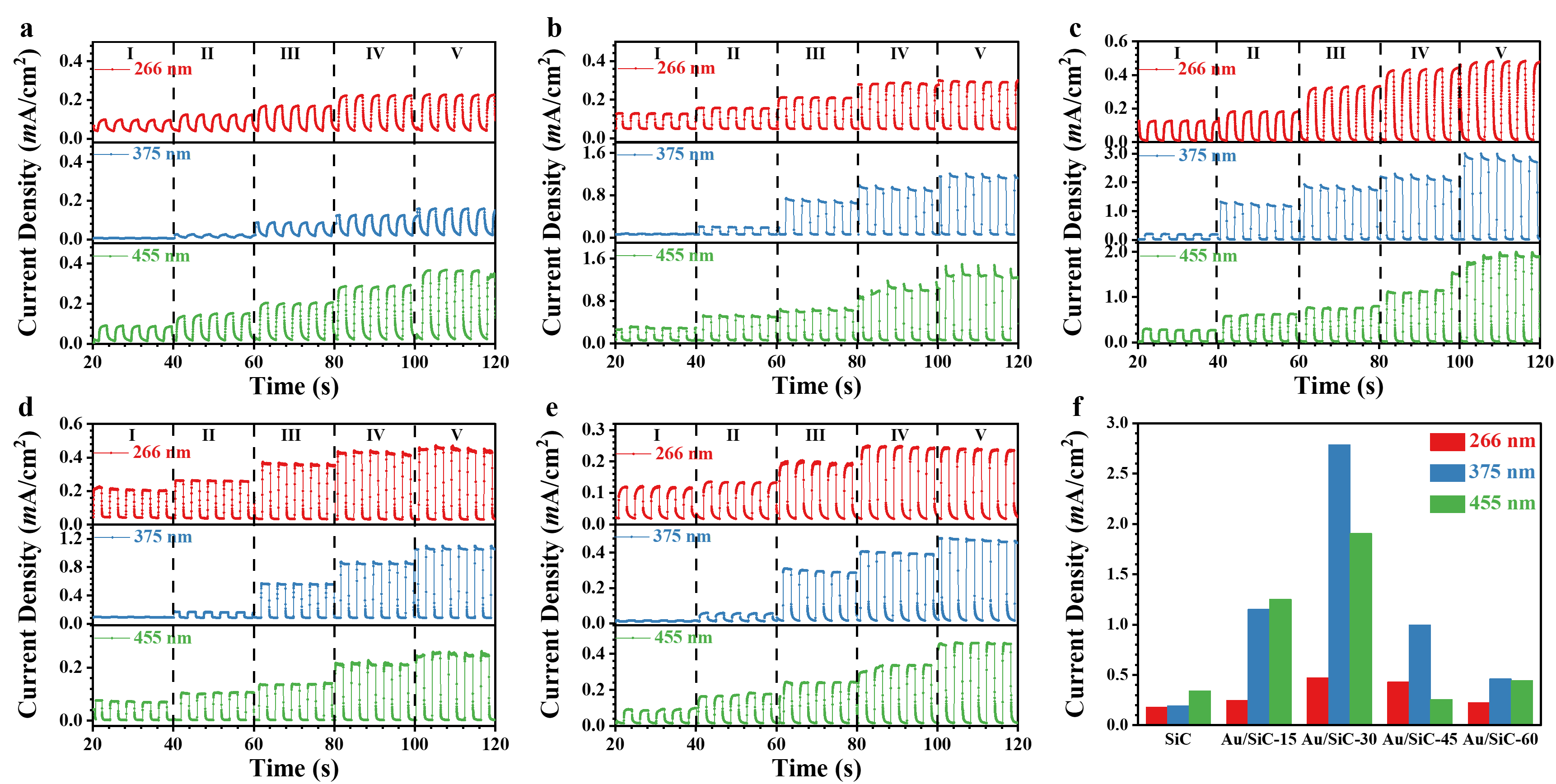
**

**Figure S4.** I-t curves of 0.5 M KOH solution with a 0.6 V bias voltage: (a) original SiC nanoarrays, (b) Au/SiC-15, (c) Au/SiC-30, (d) Au/SiC-45, (e) Au/SiC-60, respectively. (f) Photocurrent values of original SiC and various SiC/Au nanoarrays at 266 nm, 375 nm, and 455 nm.


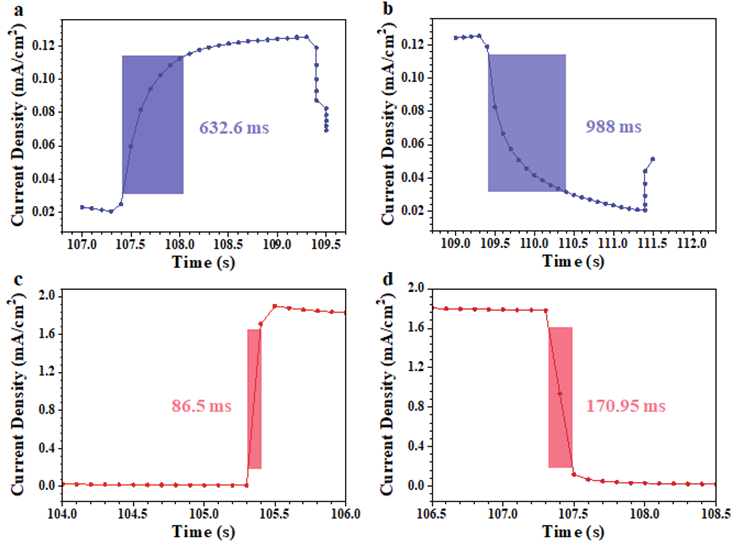


**Figure S5.** (a) Rise time and (b) decay time of original SiC nanoarrays with the bias voltage of 0.6 V. (c) Rise time and (d) decay time of SiC/Au-30 heterojunction nanoarrays with the bias voltage of 0.6 V.


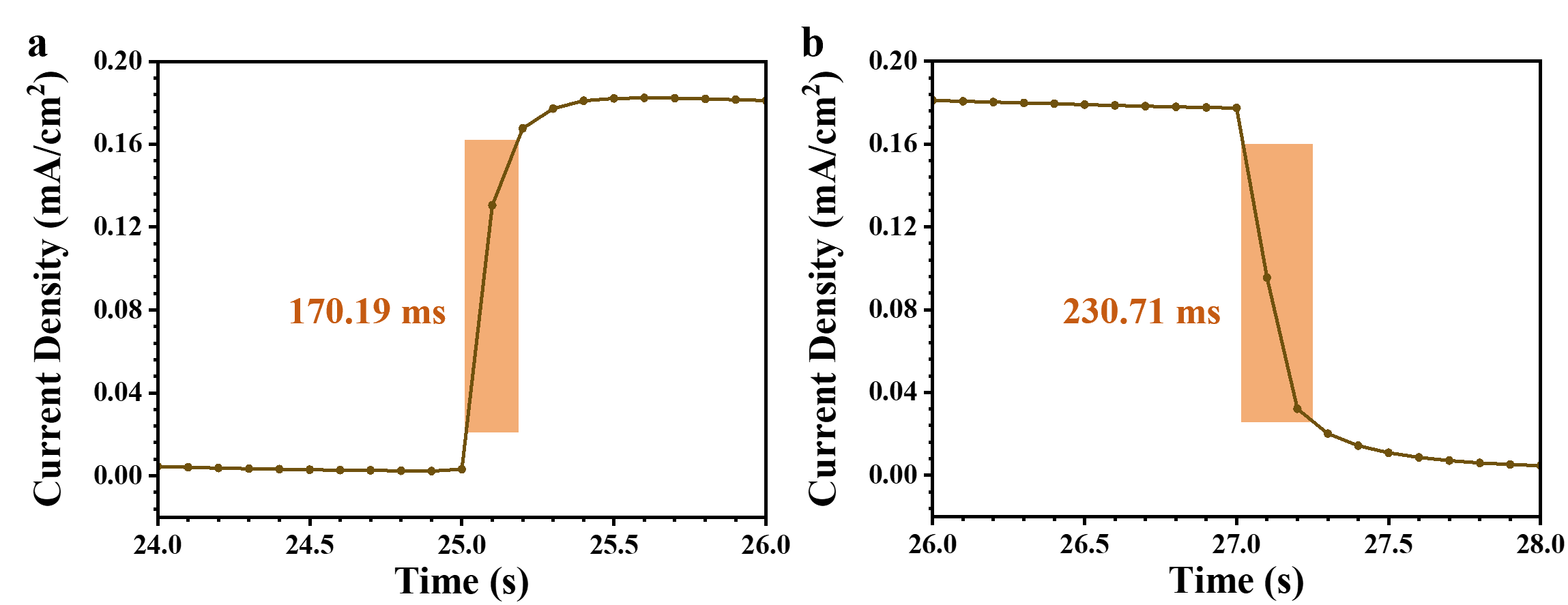


**Figure S6.** (a) Rise time and (b) decay time of SiC/Au-30 heterojunction nanoarrays with the self-powered state.


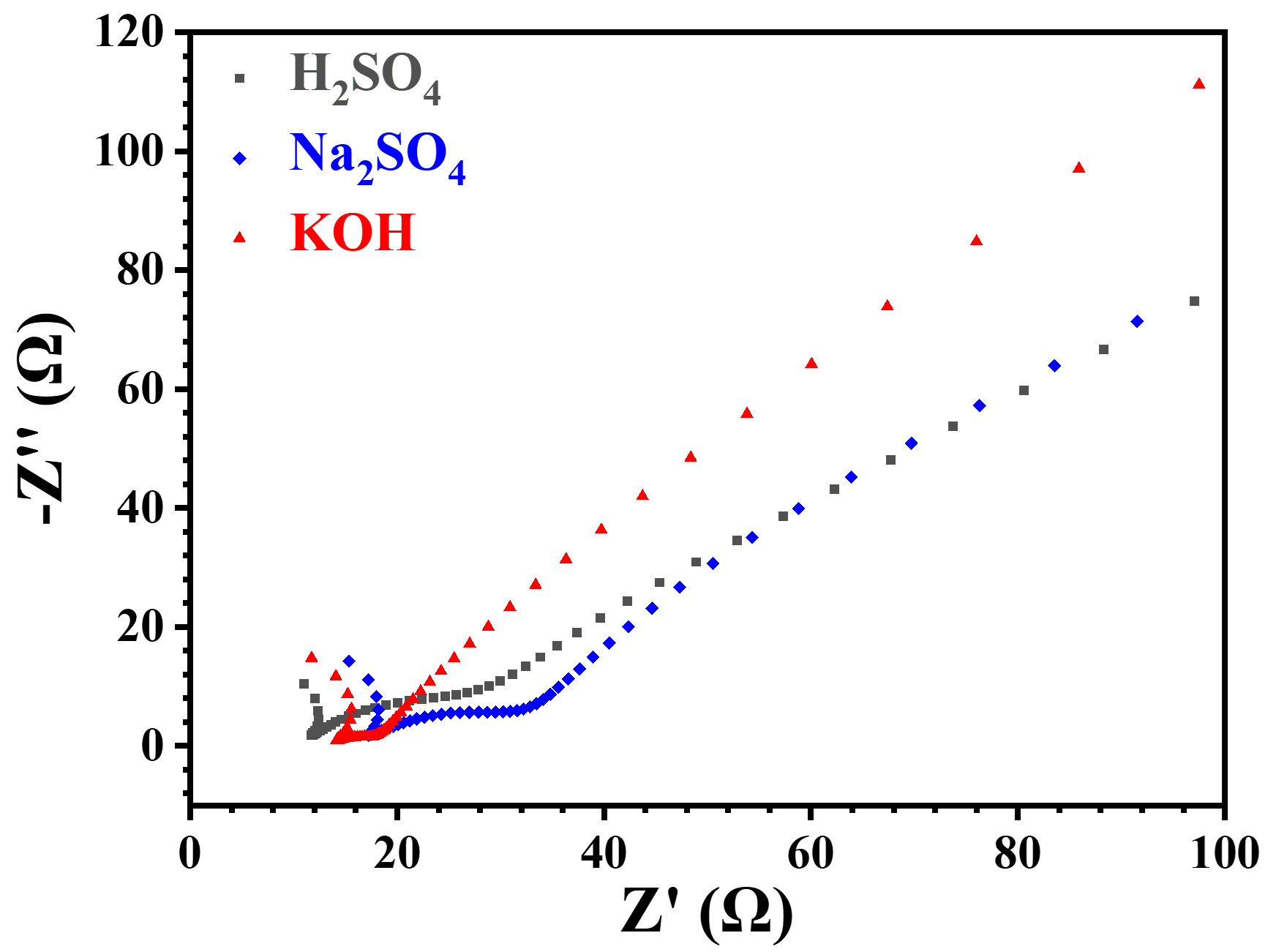


**Figure S7.** The corresponding EIS plots with 375 nm light illumination of SiC/Au-30 s samples in different solutions.


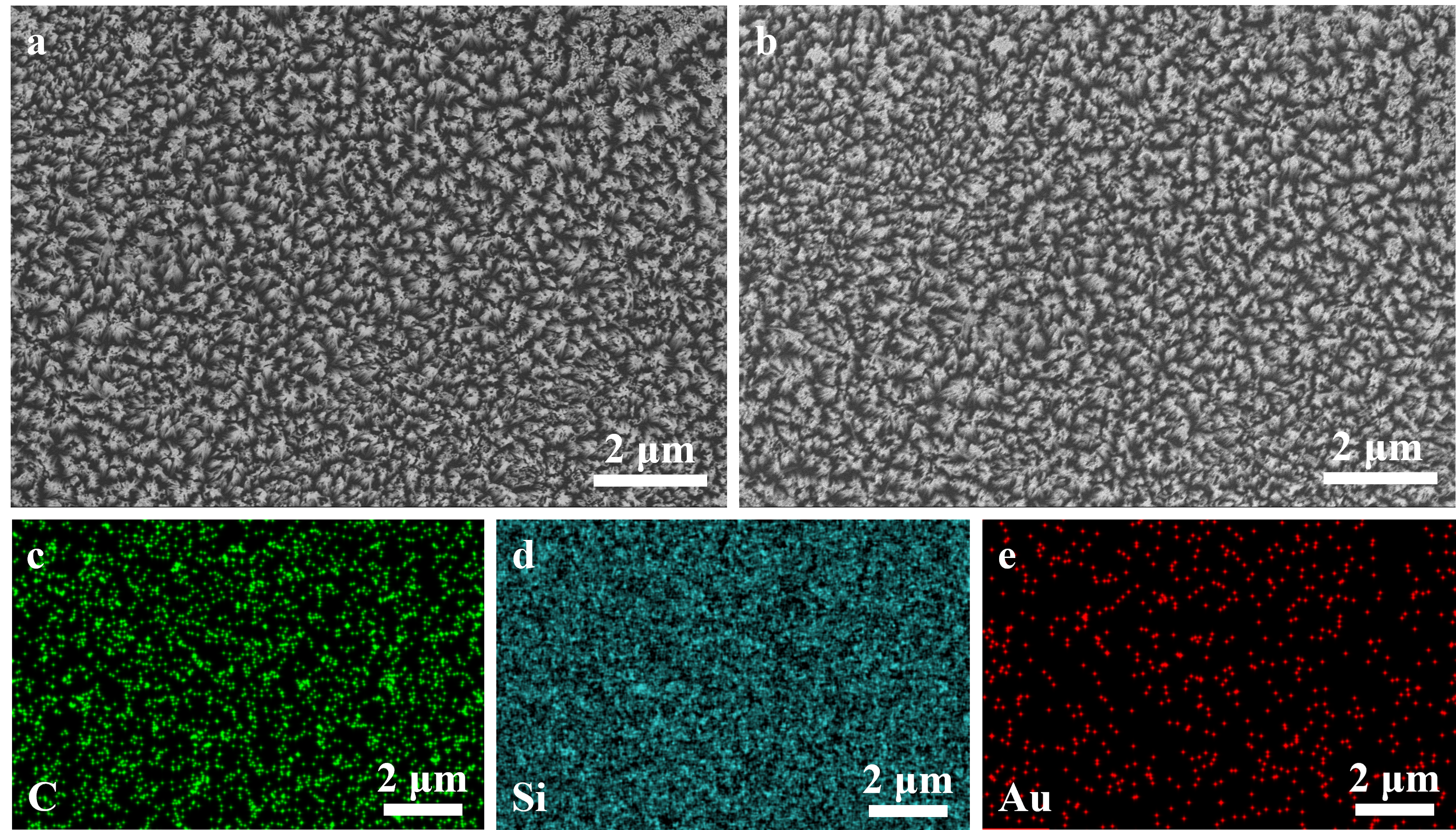


**Figure S8.** The top-view SEM images of SiC/Au-30 heterojunction nanoarrays (a) before, and (b) after the long-term stability test. The corresponding elemental mappings of (c) C, (d) Si, and (e) Au within the SiC/Au-30 heterojunction nanoarrays after the long-term stability test.
